# Methylene blue as a redox additive in electrolytes for advanced charcoal-based hybrid supercapacitors

**DOI:** 10.1038/s41598-025-22861-y

**Published:** 2025-11-06

**Authors:** Van Nhat Nguyen, An-Giang Nguyen, Thi Viet Bac Phung, Phi Long Nguyen

**Affiliations:** 1 Center for Environmental Intelligence, VinUniversity, Hanoi, 100000 Vietnam; 2 College of Engineering & Computer Science, VinUniversity, Hanoi, 100000 Vietnam

**Keywords:** Supercapacitors, Charcoal, Methylene blue, Redox electrolyte, Chemistry, Energy science and technology, Engineering, Environmental sciences, Materials science

## Abstract

Carbon-based supercapacitor electrodes derived from biomass have recently garnered significant attention due to their low cost, natural abundance, and environmental sustainability. In this study, charcoal was pretreated using a simple ultrasonic method and was employed as the active electrode material in both three-electrode and symmetric supercapacitor configurations. To further enhance electrochemical performance, a sustainable and dual-functional strategy was implemented by introducing methylene blue, a redox-active additive, into an aqueous sodium chloride electrolyte. Structural and morphological characterizations revealed that charcoal possessed a highly porous architecture with preserved plant-based vascular channels, facilitating efficient electrolyte penetration and ion transport. Electrochemical analyses demonstrated that the incorporation of methylene blue significantly enhanced charge storage through a synergistic combination of electric double-layer capacitance and pseudocapacitive behavior. The optimal device, utilizing the MB35 electrolyte composition, delivered a high specific capacitance of 212.23 F g^–1^ at 0.5 A g^–1^, an energy density of 15.34 Wh kg^–1^ at a power density of 350 W kg^–1^, and excellent cycling stability, retaining 91.3% of its initial capacitance after 2000 cycles and 84.3% after 5000 cycles at the high loading mass of 2 mg cm^− 2^. This work presents a cost-effective route for fabricating high-performance biomass-derived supercapacitors while offering a novel approach for the reutilization of dye pollutants in sustainable energy storage applications.

**Supplementary Information:**

The online version contains supplementary material available at 10.1038/s41598-025-22861-y.

## Introduction

Supercapacitors, also known as electrochemical capacitors, have attracted considerable interest in recent years due to their distinctive characteristics, including high power density, rapid charge–discharge capability, long cycle life, and excellent operational safety^1,2^. In contrast to lithium-ion batteries, which store energy through faradaic redox reactions within bulk electrode materials, supercapacitors store energy via surface-limited mechanisms, either electric double-layer capacitance (EDLC) or pseudocapacitance arising from fast surface redox reactions^3–5^. While lithium-ion batteries provide relatively high energy density, their use has been limited by slower charge–discharge rates, safety concerns, and finite cycle life^4–6^. Supercapacitors, in comparison, offer faster charge kinetics, can be recharged within seconds, and often retain performance over more than ten thousand cycles. These advantages have made supercapacitors highly suitable for applications demanding rapid energy delivery or frequent cycling, where power density, cycle stability, and safety are prioritized over energy density.

The electrochemical performance of supercapacitors has been governed primarily by the properties of electrode materials and the electrolyte. Accordingly, research efforts have focused on developing high-surface-area electrode materials with tunable porosity and surface functionalities to enhance charge storage and ion transport. Although various advanced materials, such as metal oxides, metal–organic frameworks, and conductive polymers, have exhibited promising electrochemical properties, their practical implementation has often been constrained by high synthesis costs, toxicity, and structural instability^2^. As a sustainable and cost-effective alternative, biomass-derived carbon materials have emerged as attractive candidates, owing to their natural abundance, environmental friendliness, and ease of preparation^7^. A wide range of biomass sources, including fruit peels, rice husk, leaves, etc., have been utilized to produce porous carbon materials for energy storage applications^8–10^. Among these, charcoal has been considered a particularly promising precursor due to its high carbon content, inherent porosity, and widespread availability^11^. In addition to electrode synthesis, the choice of electrolyte has played a critical role in determining the charge storage mechanism, ionic conductivity, and electrochemical window^12^. Conventional aqueous electrolytes typically serve as ion-conducting media but do not participate in redox reactions, thereby limiting total capacitance^13,14^. To address this limitation, redox-active additives such as indigo carmine, *m-*phenylenediamine, phloroglucinol, etc., have been incorporated into electrolytes to introduce pseudocapacitive contributions and enhance overall energy storage capacity^13,15,16^.

Based on the above context, charcoal was pretreated using a simple and scalable ultrasonic treatment method and was subsequently used as the active electrode in symmetric supercapacitor devices. In addition, methylene blue (MB), a widely used dye with known redox activity, was incorporated into an aqueous sodium chloride electrolyte as a redox-active additive to enhance electrochemical performance. MB has been extensively utilized in various industries, including textiles, leather, plastics, cosmetics, paper, and pharmaceuticals, and poses significant environmental risks if not properly treated. It is persistent in aquatic environments and is potentially toxic to aquatic organisms^17^. Therefore, this strategy not only improved the specific capacitance via combined EDLC and pseudocapacitive mechanisms but also provided a sustainable strategy for mitigating dye-related environmental pollution through reutilization. The findings revealed a synergistic enhancement in capacitance, energy density, and cycling stability arising from the introduction of MB into electrolyte. Electrochemical results showed that the device using the optimized electrolyte, MB35, exhibited a high specific capacitance of 212.23 F g^–1^ at a speficic current of 0.5 A g^–1^, along with an energy density of 15.34 Wh kg^–1^ at a power density of 350 W kg^–1^. These promising results demonstrate the effectiveness of using MB as a redox-active additive in conventional electrolytes and highlight the potential of biomass-derived carbon materials for high-performance and environmentally responsible supercapacitor technologies.

## Experimental

### Pretreatment of charcoal

**Fig. 1:**
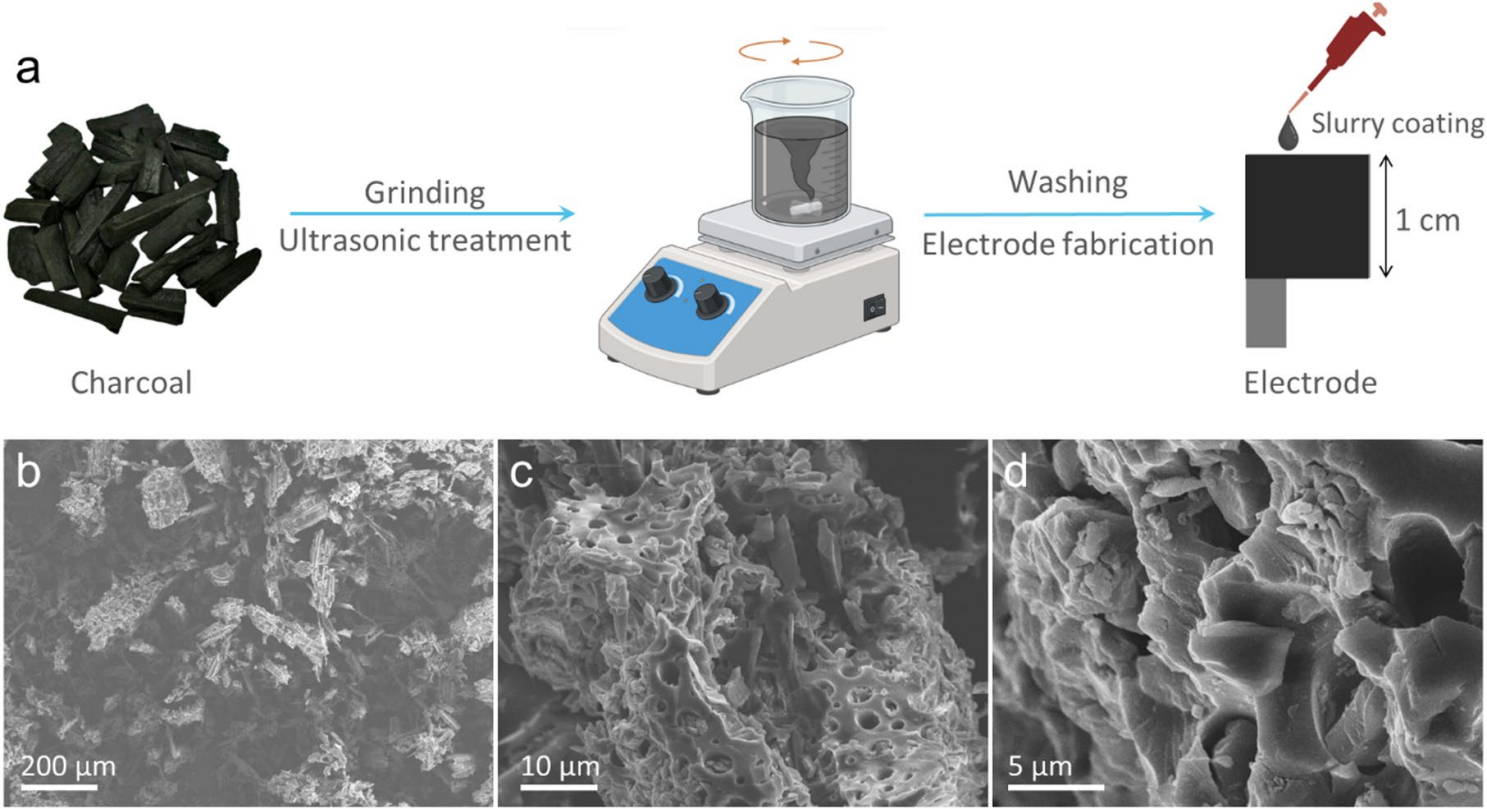
(**a**) Illustration of the preparation process of the charcoal-based electrode. (**b**–**d**) SEM images of the pretreated charcoal material at various magnifications.

Charcoal was prepared by using a simple ultrasonic process, as shown in Fig. 1. Typically, 10 g of commercial charcoal (CoalVN) was ground twice to obtain fine powder. The resulting powder was then mixed with a solution of 400 mL of ethanol and deionized (DI) water in a 1:1 volume ratio. The mixture was subjected to ultrasonication for 2 h at room temperature. After that, it was vacuum-filtered and washed several times with DI water until the pH reached 7. Subsequently, the powder was dried under vacuum at 80 °C for 12 h.

### Material characterization

The structural properties of the charcoal were investigated using a range of characterization techniques, including X-ray diffraction (XRD; SmartLab SE, Rigaku), Raman spectroscopy (XploRA ONE, Horiba), Fourier transform infrared spectroscopy (FTIR; Spectrum 400, PerkinElme), scanning electron microscopy (SEM; SU8000, Hitachi), and N_2_ adsorption–desorption analysis (HM-1208, Macsorb).

### Electrochemical characterization

The working electrode was prepared by stirring the mixture of 80 wt% of pretreated charcoal with 10 wt% of polyvinylidene difluoride (Aladdin) as a binder, and 10 wt% of black carbon (Mackalin) as a conductive agent in N-methyl-2-pyrrolidone solvent (Shanghai Zhanyun Chemical) at 60 °C for 24 h to obtain a homogeneous slurry. Then, 20 µL of the slurry was coated onto a 1 × 1 cm carbon paper substrate (Homytech, Taiwan). The coated electrode was dried under vacuum at 80 °C for 72 h. The mass loading of the electrode was controlled at approximately 1 mg cm^–2^.

The electrolyte was prepared by mixing 5 wt% sodium chloride solution (NaCl; Xilong, China) with 1 wt% methylene blue (MB; Xilong, China) at a designed ratio. Specifically, the volume ratio of NaCl: MB solution was 60:0; 55:5, 50:10, 25:35, and 0:60, and denoted as MB0, MB5, MB10, MB35, and MB60, respectively. The MB concentrations in each electrolyte was quantified by UV–Vis spectroscopy using a calibration curve in the range 0.5–20 mg L^− 1^ (**Fig. S2**) and has achieved value 0.64, 3.02, 11.95, 16.87 mg L⁻¹ for MB5, MB10, MB35 and MB60, respectively. The initial pH values of the electrolytes were 6.48 (MB0), 5.92 (MB5), 5.81 (MB10), 5.38 (MB35) and 4.69 (MB60), respectively (**Table S6**).

The three-electrode electrochemical system consisted of a working electrode, a platinum plate as the counter electrode, and an Ag/AgCl electrode as the reference electrode. The symmetric supercapacitor device was assembled by sandwiching two working electrodes with an oil-absorbing paper pre-soaked in the electrolyte, and then sealing the structure with aluminum-laminated film.

Cyclic voltammetry (CV) and Galvanostatic charge-discharge (GCD) measurements were performed at the specified scan rates or specific currents, as stated in the Results section, using an electrochemical workstation (CS310, Corrtest). Electrochemical impedance spectroscopy (EIS) was also conducted using the same workstation over a frequency range of 100 kHz to 10 mHz with an AC amplitude of 5 mV.

Specific capacitance (*C*, F g^–1^) was calculated using both CV and GCD curves. Specifically, the specific capacitances can be calculated from the CV curves (*C*_*CV*_, F g^–1^), using Eq. 1^1^:

1 $$\:{C}_{CV}\:=\:a\:\times\:\frac{\int\:IdV}{mv\varDelta\:V\:}$$

where $\:\varDelta\:V$ is potential window, defined as the difference between the ending voltage $\:{V}_{2}$ and the starting voltage $\:{V}_{1}$ in CV measurement; $\:\int\:IdV$ is the integral area under the CV curve; $\:v$ (mV s^–1^) is the scan rate; $\:m$ (g) is the mass of the active material on a working electrode; and $\:a$ is a constant, equal to 1 for a three-electrode system and 2 for a symmetric two-electrode system as the two electrodes was connected in series.

The specific capacitance was also calculated based on the GCD curves (*C*_*GCD*_, F g^–1^), using Eq. 2 for both linear and non-linear GCD behavior^1^:

2 $$\:{C}_{GCD}=a\:\times\:\frac{2I\int\:Vdt}{m\varDelta\:{V}^{2}}$$

where $\:\int\:Vdt$ is the integral area under the GCD curve, $\:I$ (A) is the current.

In the symmetrical supercapacitor device, the energy density (*E*, Wh kg^–1^) and power density (*P*, W kg^–1^) were calculated from GDC curves, as follows^1^:

3 $$\:E=\frac{I\int\:Vdt}{3.6m}$$

4 $$\:P=\frac{E\times\:3600}{t}$$

where $\:t$ is the discharge time.

## Results and discussion

### Structural and morphological properties of electrode material

Charcoal was pretreated through a straightforward ultrasonic treatment, as illustrated in Fig. 1a. The structure and morphology of pretreated charcoal were investigated by SEM. As shown in Fig. 1b–d, the sample exhibited a porous microstructure characterized by an interconnected network of pores and channels. This complex structure originally came from the plant’s xylem vessels, which are responsible for transporting water and nutrients from the roots to the leaves. Notably, this natural structure was preserved during the carbonization process, resulting in a highly porous charcoal material. Such a structure is advantageous for electrolyte penetration and may facilitate efficient ion transport within the electrode^18,19^.

**Fig. 2:**
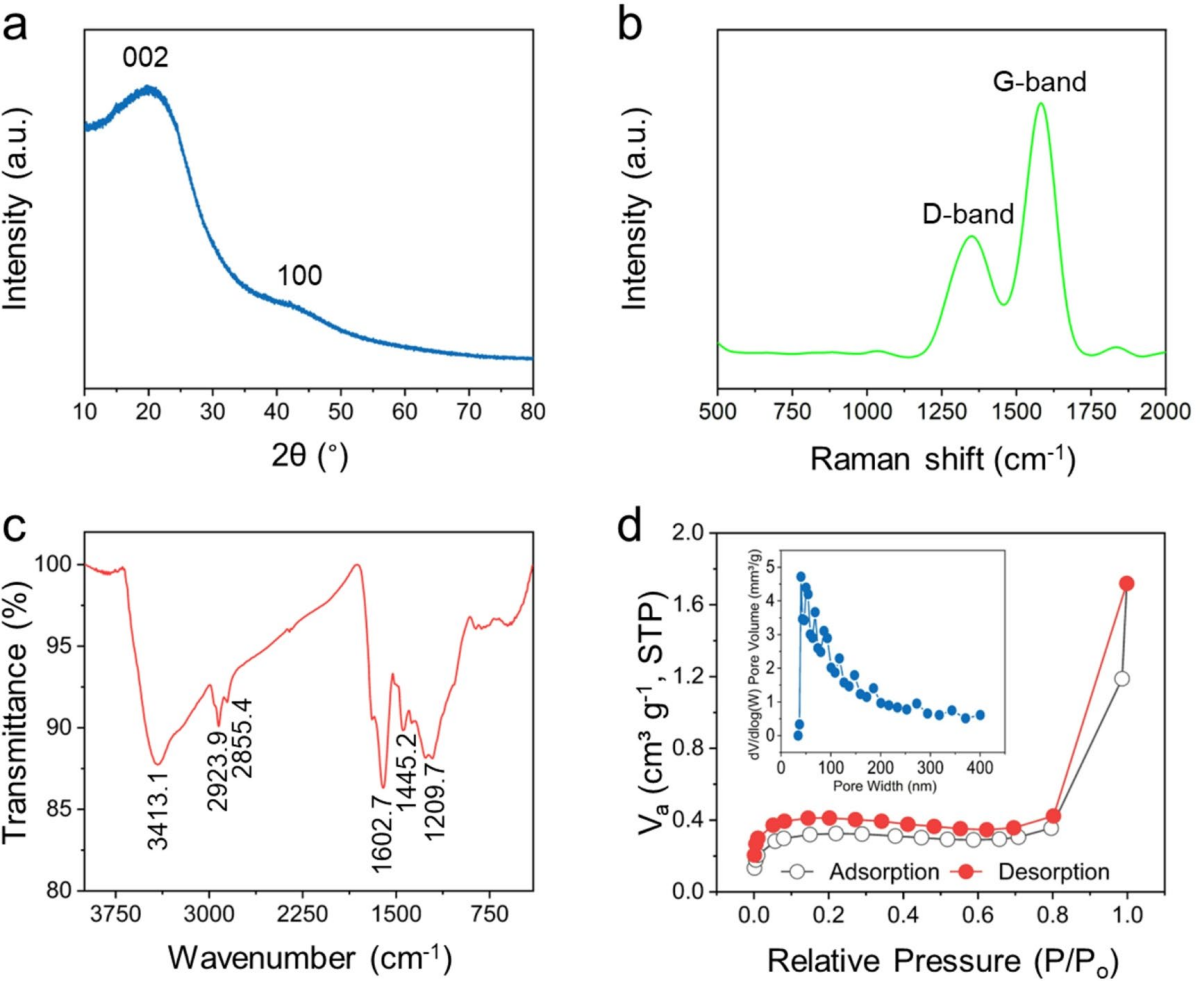
(**a**) XRD pattern; (**b**) Raman spectrum; (**c**) FTIR spectrum; and (**d**) N_2_ adsorption–desorption isotherm of the charcoal material.

The structure of charcoal was investigated by XRD, as shown in Fig. 2a. Typically, the sample has two broad peaks at 20.4° and 42.7°, which indicate the turbostratic carbon structure between amorphous and crystalline carbon. In addition, these peaks can be assigned to *(002)* and *(100)* planes, respectively, which correspond to the stacking of graphitic layers and the in-plane ordering within the graphitic layers^3,10,20^. To confirm the turbostratic nature of the synthesized carbon, Raman analysis was conducted. As presented in Fig. 2b, two characteristic bands were observed: the *D-*band at 1349 cm^–1^ and the *G-*band at 1582 cm^–1^. The *D-*band is associated with structural defects in the graphite layers, while the *G-*band corresponds to the *E*_*2g*_ phonon mode arising from the in-plane stretching vibration of *sp*^*2*^*-*hybridized carbon atoms^9,21,22^. The intensity ratio of the *D-*band to the *G-*band (*I*_*D*_*/I*_*G*_) was approximately 0.47, indicating that the sample was predominantly graphitic with a relatively low level of disorder. In addition, FTIR spectrum revealed the surface functional groups of the electrode materials, as displayed in Fig. 2c. A broad and intense absorption band at 3413 cm^–1^ was attributed to the O–H stretching vibration, which may arise from physically adsorbed water molecules or hydroxyl groups present on the charcoal surface. The peaks at 2923 and 2855 cm^–1^ corresponded to symmetric and asymmetric C–H stretching vibrations, respectively. A peak near 1602 cm^–1^ was assigned to the C = C stretching mode, typically associated with carbonyl-containing functional groups^11^. Additionally, the bands observed at 1446 cm^–1^ and 1209 cm^–1^ were attributed to vibrational modes related to carboxyl groups^11^. The N_2_ adsorption–desorption isotherms of charcoal exhibited a type II profile, indicating the presence of a predominantly macroporous structure. In addition, the specific surface area of the charcoal was determined to be approximately 1.29 m^2^ g^–1^. Such low BET values are typical for biochars rich in macropores, as N_2_-BET often underestimates their accessible surface area^28^. The pore size distribution, calculated using the Barrett–Joyner–Halenda method, was presented in the insets of Fig. 2d. Most of the macropores in the charcoal were found to fall within the range of 100–200 nm. Such a macroporous structure is advantageous for facilitating electrolyte diffusion and improving ion transport pathways, thereby may enhance the accessibility of the electrode surface for charge storage.

### Electrochemical characterization using a three-electrode system

**Fig. 3:**
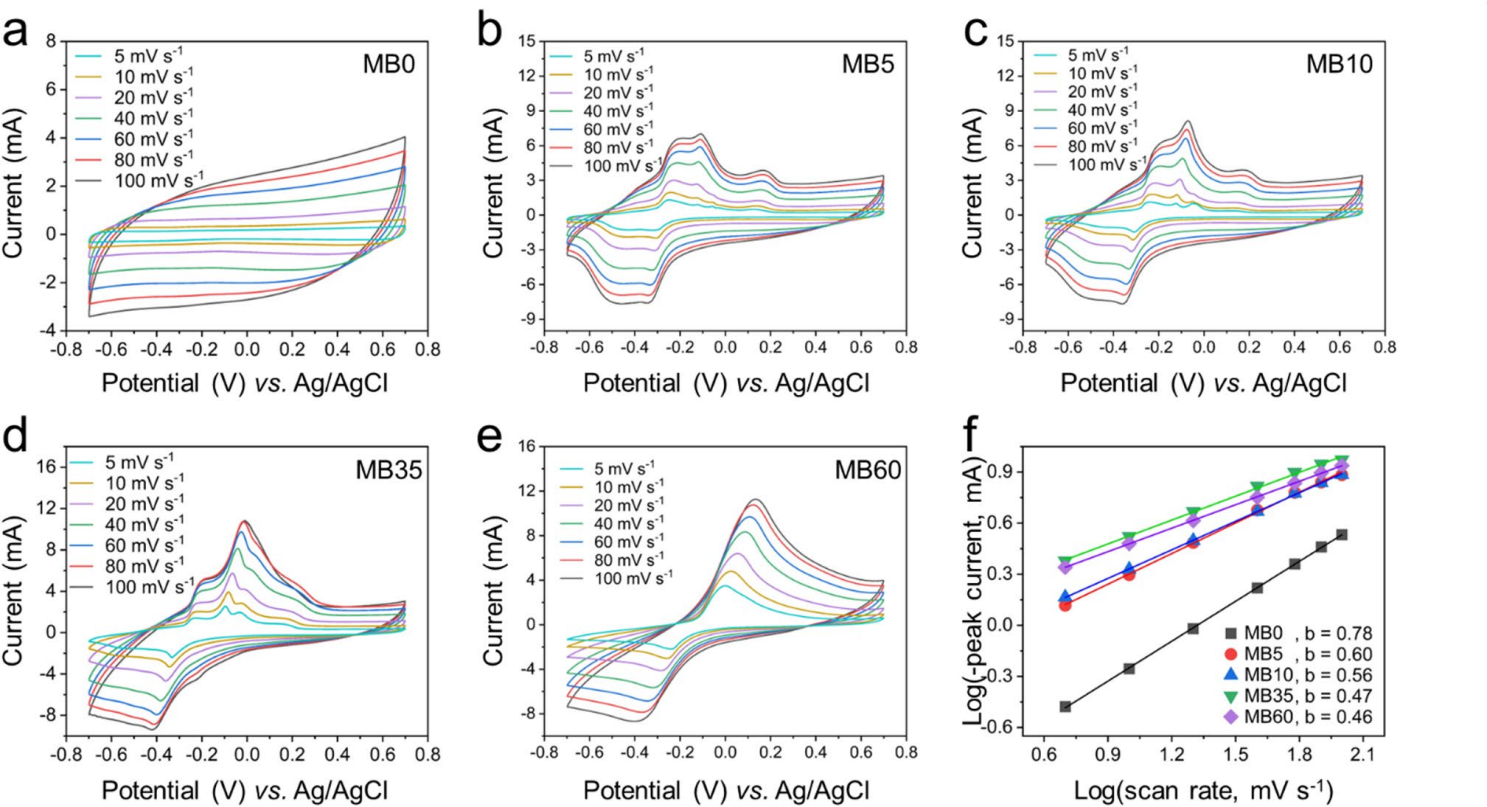
CV curves at various scan rates for the three-electrode system: (**a**) MB0, (**b**) MB5, (**c**) MB10, (**d**) MB35, and (**e**) MB60. (**f**) Logarithmic plots of peak current vs. scan rate and the corresponding *b-*values.

The electrochemical properties of charcoal were initially evaluated in a three-electrode configuration. CV measurements were conducted over a potential window of − 0.7 V to 0.7 V vs. Ag/AgCl at scan rates ranging from 5 to 100 mV s^–1^ in various electrolytes, including MB0, MB5, MB10, MB35, and MB60, as shown in Fig. 3. In the MB0 electrolyte, without MB addition, the CV curves exhibited a nearly rectangular shape at low scan rates, characteristic of EDLC behavior. However, as the scan rate increased, the CV profiles became increasingly elliptical, indicating the influence of internal resistance on ion transport and charge storage^23^. These observations suggest that the capacitance in the MB0 system is primarily governed by EDLC mechanisms^24^. In contrast, for electrolytes containing MB, MB5 to MB60, which represent increasing concentrations of the redox-active additive, distinct redox peaks emerged in the CV curves. Specifically, a cathodic peak appeared between − 0.5 V and − 0.3 V, while an anodic peak was observed in the range of − 0.2 V to 0.2 V. These redox features are attributed to the reversible redox reactions of MB molecules, confirming the pseudocapacitive contribution introduced by the MB additive^25^. Moreover, compared to the MB0 electrolyte, the current response associated with the redox peaks increased significantly in the MB5–MB60 systems and reached a maximum value of approximately 10.8 mA in the MB35 and MB60 electrolytes. As the scan rate increased, the redox peaks became more prominent and shifted toward regions of higher absolute potential. This shift, coupled with the increase in peak intensity, is indicative of a pseudocapacitive behavior governed by fast surface redox reactions^26^. For comparison, the CV of five systems at 100 mV s^–1^ was presented in Fig. S1. The MB0 sample exhibited the smallest CV curve area, indicating the lowest specific capacitance among the five tested samples. The specific capacitances were calculated from the CV curves using Eq. 1 and listed in **Table ****S1**. Upon the addition of the redox-active agent MB, the CV curve areas of MB5, MB10, and MB35 increased progressively with increasing MB concentration. The specific capacitance incresed from 46.45 F g^–1^ of MB0 to 75.51 F g^–1^ of MB35 at 100 mV s^–1^. This enhancement is attributed to the combined contribution of EDLC and pseudocapacitance arising from the redox activity of MB. It is also noteworthy that increasing the MB concentration from MB5 to MB60 resulted in greater polarization. Although the MB60 system exhibited the largest CV area, indicating the highest specific capacitance of 83.50 F g^–1^ at 100 mV s^–1^, it also showed the most pronounced polarization. This behavior is likely attributed to an excessive concentration of redox-active species, which can hinder ion transport and introduce diffusion limitations. Based on these observations, the MB35 electrolyte appears to provide an optimal balance between enhanced pseudocapacitance and manageable polarization, making it the most promising candidate among the tested systems.

To clarify the storage mechanism of these systems, the power-law relationship between the peak current (*I*, mA) and scan rate ($\:v$, mV s^–1^) was employed, as described by the following equation^22^:

5 $$\:I\:=\:a{v}^{b}$$

By taking the logarithm of both sides, Eq. 5 can be rewritten as:

6 $$\:\text{log}\left(I\right)=\text{l}\text{o}\text{g}\left(a\right)+blog\left(v\right)$$

where $\:a$ and $\:b$ are constants. If the plot of log*(I) vs.* log*(*$\:v$*)* yields a linear relationship, the slope corresponds to the *b-*value, which provides insights into the underlying charge storage mechanism. It is generally accepted that a *b-*value of 1 indicates a surface-controlled process typical of an EDLC mechanism, while a *b-*value of 0.5 suggests a diffusion-controlled process associated with pseudocapacitance involving redox reactions^27^. As shown in Fig. 3f, all samples exhibited a linear log*(I) –* log*(*$\:v$*)* relationship, confirming the applicability of the power-law model. The *b-*value for the cell using MB0 electrolyte was 0.78, indicating that the charge storage was predominantly governed by EDLC behavior. In contrast, the *b-*values for MB5, MB10, MB35, and MB60 electrolytes were 0.60, 0.56, 0.47, and 0.46, respectively, suggesting a transition toward a pseudocapacitive mechanism dominated by redox reactions of MB.

To get a more complete view of the capacitive contribution ratio of each mechanism, we used Dunn’s method for capacitive–diffusive contribution analysis with Eq. 7 and Eq. 8 as follows^22^:

7 $$\:i\left(V\right)={k}_{1}v+{k}_{2}{v}^{1/2}$$

8 $$\:\frac{i\left(V\right)}{{v}^{1/2}}={k}_{1}{v}^{1/2}+{k}_{2}$$

where $\:v$ (mV s^–1^) is the scan rate, and the coefficients $\:{k}_{1}$ and $\:{k}_{2}$ are constants obtained from the linear fit line of $\:\frac{i\left(V\right)}{{v}^{1/2}}$ with $\:{v}^{1/2}$ to help determine the ratio of each mechanism’s contribution to the total individual capacitance. The capacitive–diffusive contribution ratios of MB0, MB35, and MB60 were shown in **Fig. S3**. Dunn analysis showed that the diffusive mechanism became increasingly dominant as the MB concentration increased, with contributions of 32.7%, 91.5%, and 95.4% at the scan rate of 100 mV s^− 1^ for MB0, MB35, and MB60, respectively. This suggests that the increase in specific capacitance of MB35 and MB60 samples was mainly due to the participation of the redox reaction of MB.

**Fig. 4:**
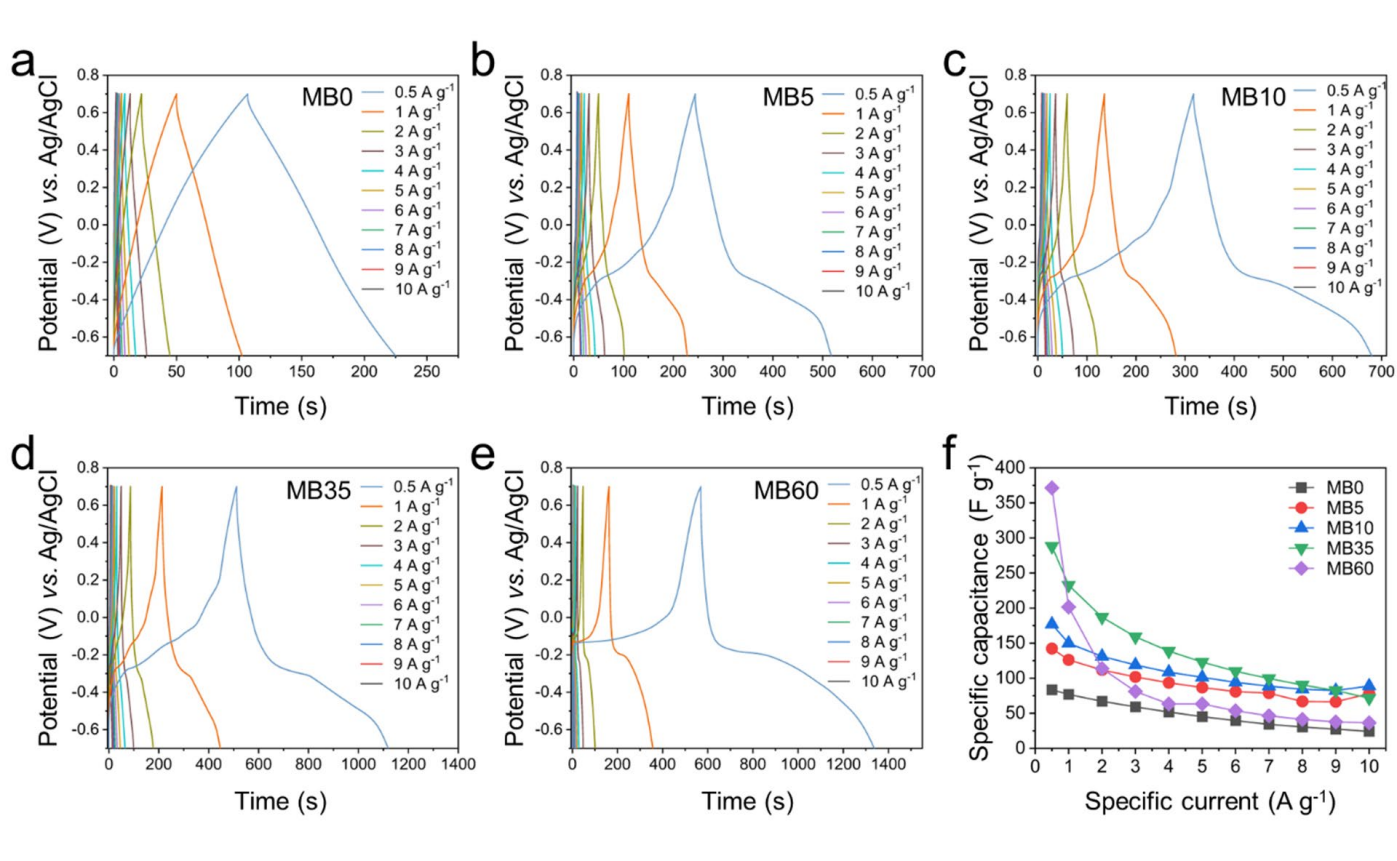
Galvanostatic charge–discharge curves at various specific currents for the three-electrode system: (**a**) MB0, (**b**) MB5, (**c**) MB10, (**d**) MB35, and (**e**) MB60. (**f**) Comparison of specific capacitances of all samples at various specific currents in the three-electrode system.

To further investigate the electrochemical performance of the MB0, MB5, MB10, MB35, and MB60 systems, GCD tests were conducted within a potential window of − 0.7 V to 0.7 V vs. Ag/AgCl at specific currents ranging from 0.5 to 10 A g^–1^. As shown in Fig. 4, the GCD curve of the cell using the MB0 electrolyte displayed a nearly linear profile with a symmetric isosceles triangular shape, characteristic of EDLC behavior. In contrast, the GCD curves of the MB5, MB10, MB35, and MB60 samples exhibited distinct voltage plateaus, which are attributed to redox reactions involving MB, consistent with the CV results. Notably, the discharge time increased proportionally with MB concentration across all specific currents, which implies the higher specific capacitance can be obtained. The GCD data was used to calculate specific capacitances based on Eq. 2, with the results presented in Fig. 4f and **Table S2**. At a specific current of 0.5 A g^–1^, the MB0, MB5, MB10, and MB35 systems delivered specific capacitances of 83.59, 142.26, 177.05, and 288.08 F g^–1^, respectively. To eliminate the specific capacitance contribution from the carbon substrate, the electrochemical properties (CV, GCD) of the carbon substrate were investigated under the same conditions as the electrolyte solution of MB0 as shown in **Fig. S4**. The specific capacitance of the carbon substrate only reached about 16.4 F g^− 1^ at the specific current of 0.5 A g^–1^, which accounted for about 19.6% of the specific capacitance of MB0. Interestingly, the MB60 system exhibited the highest specific capacitance of 371.45 F g^–1^ under the same conditions. As expected, the specific capacitance decreased with increasing specific current. This reduction is commonly attributed to the shorter charge–discharge time at higher specific currents, which limits the ability of electrolyte ions to fully penetrate the internal pores of the electrode. Instead, ions can only access the outer surface of the porous structure, resulting in partial utilization of the electrode material and lower overall capacitance^10^. Among all samples, the MB35 system demonstrated the most balanced performance, delivering specific capacitances of 288.08, 232.79, 187.08, 159.07, 138.67, 123.10, 109.84, 99.68, 90.35, 82.48, and 71.62 F g^–1^ at 0.5, 1, 2, 3, 4, 5, 6, 7, 8, 9, and 10 A g^–1^, respectively. Although the MB60 sample showed the highest capacitance of 371.45 F g^–1^ at low specific current of 0.5 A g^–1^, its performance declined significantly at higher specific currents, eventually approaching the values observed for MB0. These findings further support the argument that MB35 provides optimal performance, making it the most effective electrolyte composition in this study.

### Electrochemical characterization of symmetric supercapacitor devices

**Fig. 5:**
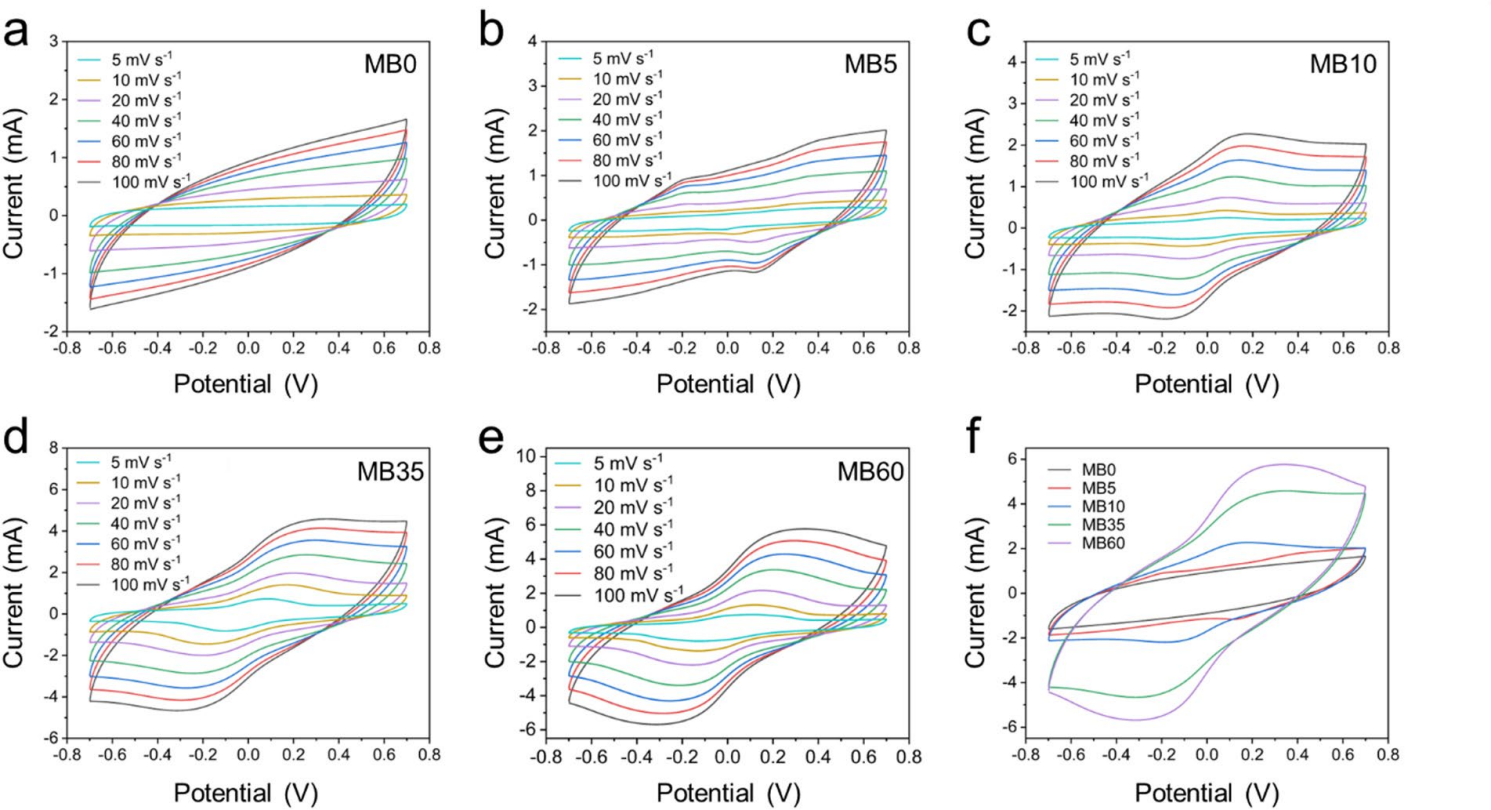
CV curves at various scan rates for the symmetric device: (**a**) MB0, (**b**) MB5, (**c**) MB10, (**d**) MB35, and (**e**) MB60. (**f**) Comparison of CV curves of all samples at a scan rate of 100 mV s^–1^.

The electrochemical performance of the charcoal electrode with different electrolytes was further evaluated in a symmetric supercapacitor configuration. CV profiles were recorded at scan rates ranging from 5 mV s^–1^ to 100 mV s^–1^ within a potential window of − 0.7 V to 0.7 V for devices assembled with MB0, MB5, MB10, MB35, and MB60 electrolytes, as shown in Fig. 5. Consistent with the results from the three-electrode system, the CV curve of the device using the MB0 electrolyte exhibited a near-rectangular shape, indicative of ideal electric EDLC behavior^10^. In contrast, devices with MB5, MB10, MB35, and MB60 electrolytes showed distinct redox peaks, attributed to the redox activity of MB. The addition of MB transformed the dominant charge storage mechanism from EDLC to a hybrid of EDLC and pseudocapacitance, thereby enhancing the overall charge storage capability. These findings align with the results presented in Section "**Electrochemical characterization using a three-electrode system"**. CV curves at 100 mV s^–1^ for MB0 to MB60 devices were displayed in Fig. 5f, where the increasing CV area correlates with the increasing MB concentration and enhanced redox activity, leading to improved specific capacitance, as calculated by Eq. 1. Specifically, devices with MB0, MB5, MB10, MB35, and MB60 electrolytes delivered specific capacitances of 110.48, 123.95, 134.92, 292.74, and 317.27 F g^–1^ at 5 mV s^–1^, respectively (**Table S3**).

**Fig. 6:**
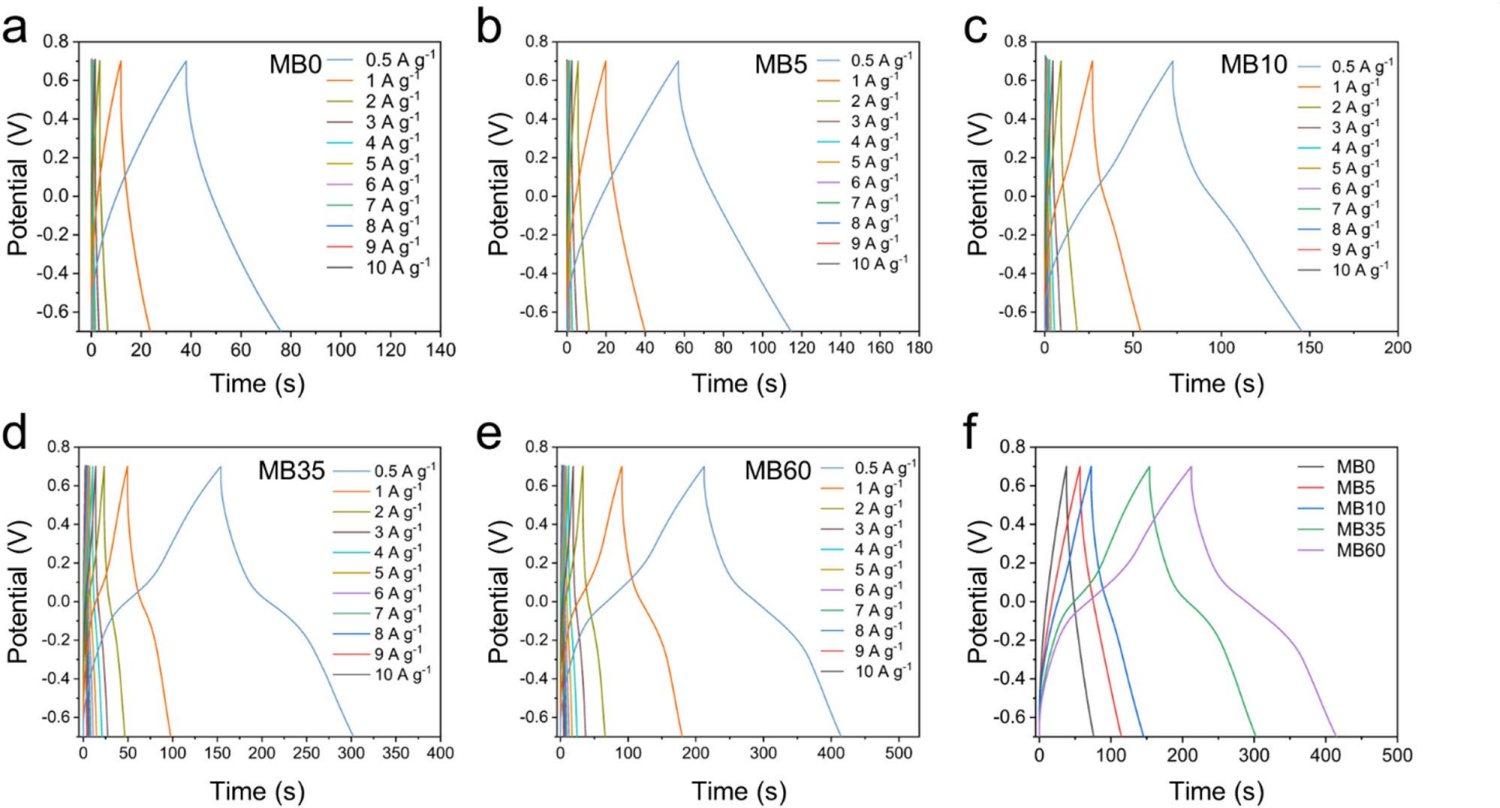
Galvanostatic charge–discharge curves at various specific currents for the symmetric device: (**a**) MB0, (**b**) MB5, (**c**) MB10, (**d**) MB35, and (**e**) MB60. (**f**) Comparison of specific capacitances of all samples at various specific currents in the symmetric device.

**Fig. 7:**
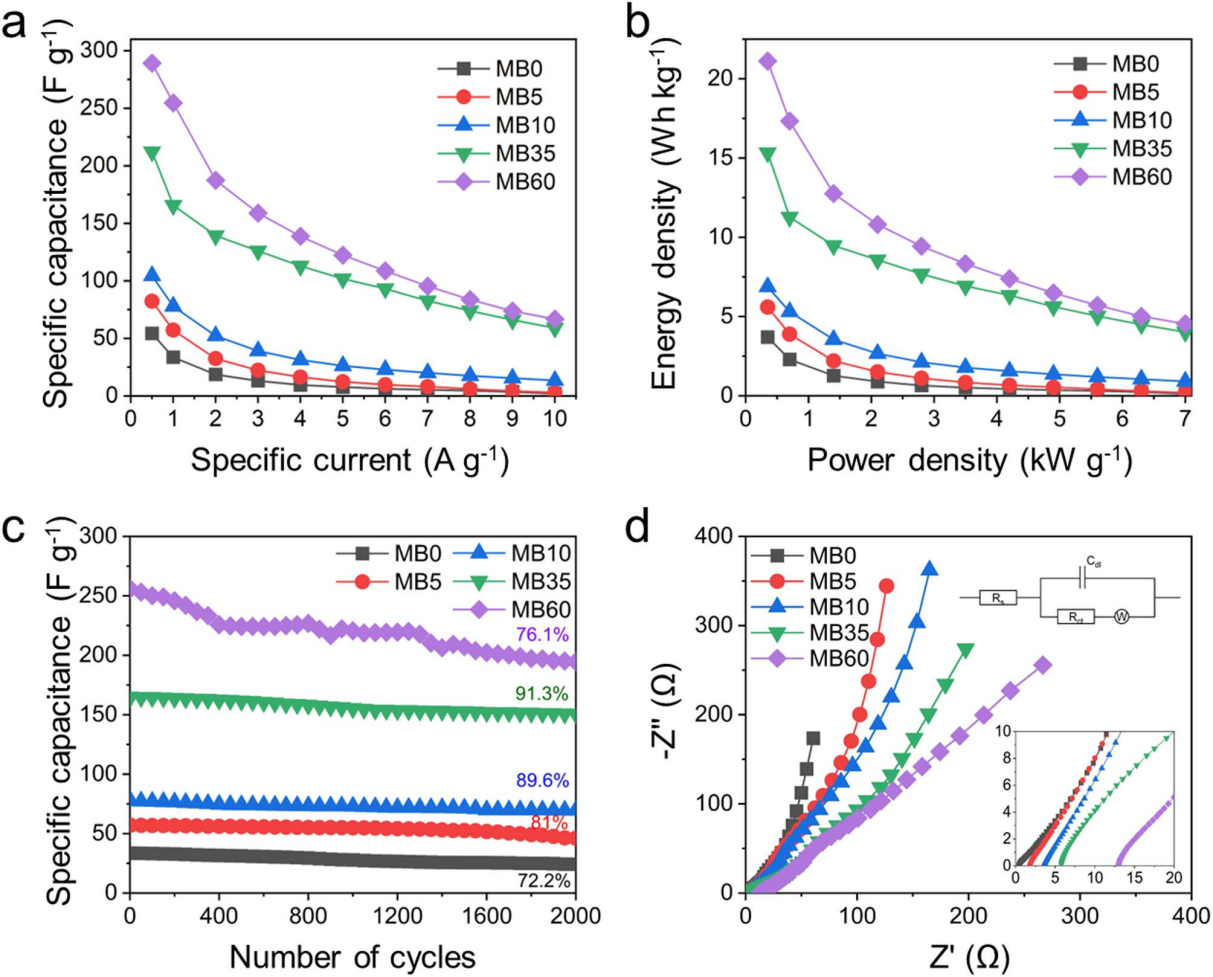
(**a**) Specific capacitances at various specific currents for the symmetric devices. (**b**) Ragone plots. (**c**) Cycling stability of the MB0, MB5, MB10, MB35, and MB60 devices at 1.0 A g^–1^. (**d**) Nyquist plots with inset showing the high-frequency region and the equivalent circuit used for fitting.

GCD measurements were also conducted on the symmetric devices at specific currents ranging from 0.5 to 10 A g^–1^ within the same voltage window (Fig. 6). Similar to the CV data, the GCD curves exhibited two distinct features: linearity and non-linearity. For the MB0 device, the charge–discharge profile was nearly linear, characteristic of EDLC behavior (Fig. 6a). As the MB concentration increased in MB5, MB10, MB35, and MB60 devices, the GCD curves became progressively less linear, indicating increasing pseudocapacitive contributions. Notably, for the MB10, MB35, and MB60 devices, which contained sufficient concentrations of MB, the GCD curves were fully non-linear. These devices thus exhibit hybrid capacitance, combining both EDLC and pseudocapacitive mechanisms. The specific capacitance values, calculated at various specific currents using Eq. 2, were shown in Fig. 7a and **Table S4**. Devices using MB5, MB10, MB35, and MB60 electrolytes exhibited significantly higher capacitance than MB0 across all specific currents. Notably, at 0.5 A g^–1^, the MB35-based device achieved a specific capacitance of 212.23 F g^–1^, nearly four times higher than that of MB0 (54.29 F g^–1^).

Ragone plots were also constructed using the data obtained from the GCD tests. As shown in Fig. 7b, the energy density of the devices increased with the addition of MB at each given power density. At a power density of 350 W kg^–1^, the energy density values of the MB0, MB5, MB10, MB35, and MB60 devices increased progressively to 3.70, 5.60, 6.87, 15.34, and 21.11 Wh kg^–1^, respectively, indicating the beneficial effect of redox-active MB on charge storage capability. In particular, the MB35-based symmetric supercapacitor exhibited an energy density of 15.34 Wh kg^–1^, which is approximately four times higher than that of the MB0 device (3.70 W h kg^–1^) at the same power density. These enhancements in electrochemical performance are primarily attributed to the pseudocapacitive contributions introduced by MB, as discussed earlier. Interestingly, the MB60 device outperformed MB35 in both GCD and Ragone plots, a trend that contrasts with the behavior observed in the three-electrode configuration. This discrepancy may result from the limited volume of electrolyte in the symmetric cell, which likely results in a thinner MB-derived layer on the electrode surface compared to the excess MB in the three-electrode setup. However, at specific currents above 7 A g^–1^, the specific capacitance and energy density values of the MB35 and MB60 devices became comparable. The long-term cycling stability of all devices was also evaluated at a specific current of 1.0 A g^–1^ (Fig. 7c). The initial specific capacitances of the MB0, MB5, MB10, MB5, and MB60 devices were measured as 33.61, 57.01, 77.61, 164.90, and 255.80 F g^–1^, respectively. After 2000 cycles, these values were retained at 24.27, 46.16, 69.53, 150.62 and 194.68 F g^–1^, corresponding to capacitance retention rates of 72.2%, 81.0%, 89.6%, 91.3%, and 76.1%, respectively. These results indicate that the MB35-based device exhibited superior cycling stability compared to the other systems. Considering both specific capacitance and long-term stability, the MB35 device exhibited the most balanced electrochemical performance among all tested systems. For further comparison, the performance of the MB35-based device was benchmarked against other carbon-based supercapacitors, as summarized in Table 1.

**Table 1:** Comparison of the electrochemical performance of supercapacitors using redox-active additives in the electrolyte.

Electrode material	Electrolyte	Potential window (V)	Specific capacitance (F g^–1^)	Cycling stability @ retention	Refs.
Multiwalled carbon nanotubes	H_2_SO_4_/Indigo Carmine	0–1	50	10,000 @ 70%	^13^
Activated carbon extracted from natural bio-waste honeycomb	PVDF-HFP/EC: PC-NaBF_4_/MB	0–1.4	114 @ 1.0 A g^–1^	10,000 @ 64%	^14^
Activated carbon	KOH/m-phenylenediamine	− 0.5 to 0.5 V	78,01 @ 0.5 A g^–1^	10,000 @ 90,68%	^15^
Rice husk	Na_2_SO_4_/K_3_Fe[CN]_6_	0–0.8	188 @ 0.5 A g^–1^	5000 @ 97.7%	^20^
Multiwalled carbon nanotubes	H_2_SO_4_/MB	0–0.104	23	6000 @ 88%	^25^
Charcoal	MB35	− 0.7 to 0.7 V	212.23 @ 0.5 A g^–1^ 165.62 @ 1.0 A g^–1^	2000 @ 91.3%	This work

EIS analysis was conducted to further investigate the interfacial behavior of the symmetric supercapacitor devices after cycling. As shown in Fig. 7d, the obtained Nyquist plots consist of a semicircle in the high-frequency region, followed by a sloped linear segment in the low-frequency region. These features are characteristic of charge-transfer processes and ion diffusion behavior, respectively. To analyze these plots, an equivalent electrical circuit model was employed. In this model, *R*_*s*_ represents the internal resistance of the system, including electrode and electrolyte resistance, *R*_*ct*_ corresponds to the charge-transfer resistance at the electrode–electrolyte interface, C_dl_ denotes the double-layer capacitance, and W represents the Warburg impedance, which reflects ion diffusion in the porous electrode material. The fitting values from the fitted model are summarized in Table 2. The *R*_*s*_ of MB0, MB5, MB10, MB35, and MB60 were 0.45, 1.83, 3.61, 5.93, and 13.23 Ω, respectively. In addition, the *R*_*ct*_ were 0.67, 0.65, 0.52, 2.78, and 2.66 Ω, respectively. These results indicate that the addition of MB led to an increase in both series resistance and charge-transfer resistance, particularly for the MB60 electrolyte. This increase is likely due to the formation of a thicker MB-derived film on the electrode surface, which may hinder ion transport and electron transfer at the interface.

**Table 2:** EIS fitting results from Nyquist plots of supercapacitor devices after cycling.

Element	MB0	MB5	MB10	MB35	MB60
R_s_ (Ω)	0.45	1.83	3.61	5.93	13.23
R_ct_ (Ω)	0.67	0.65	0.52	2.78	2.66
C_dl_ (µF)	90.24	140.23	129.46	71.53	59.46
Warburg	24.10	38.06	43.00	89.84	71.26

**Fig. 8:**
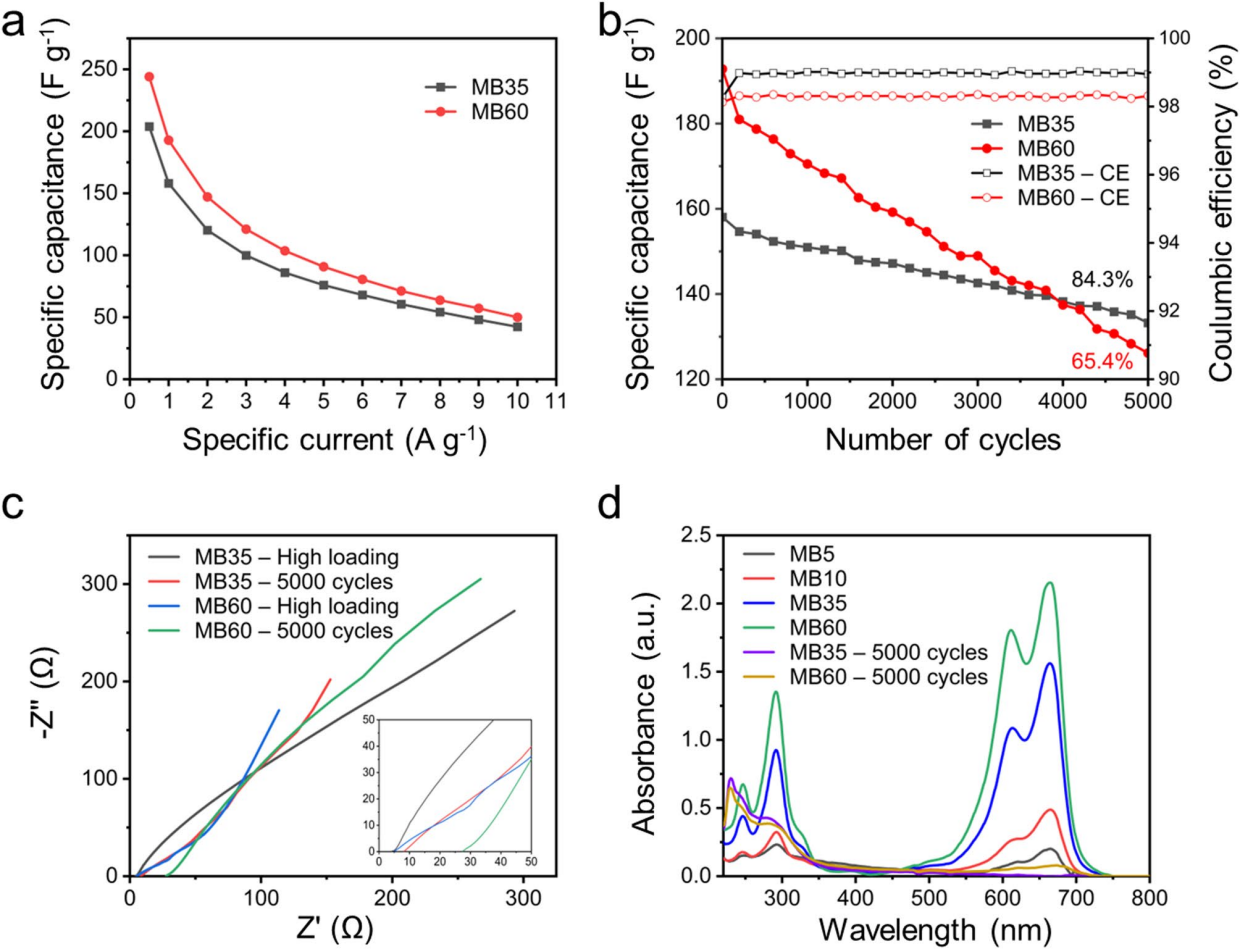
(**a**) Specific capacitances at various specific currents for MB35 and MB60 at the mass loading of 2 mg cm^-2^. (**b**) Cycling stability of the MB35, and MB60 devices at 1.0 A g^–1^ at the mass loading of 2 mg cm^-2^. (**c**) Nyquist plots. (**d**) Evolution of the UV–Vis absorption spectra of the electrolyte solutions before and after 5000 cycles.

To evaluate the scalability and practical application of Charcoal-based electrode materials, we investigated the electrochemical performance at higher mass (2 mg cm^− 2^). The specific capacitance of MB35 and MB60 devices still reached high specific capacitance values ​​of approximately 203.84 and 244.08 F g^− 1^ at 0.5 A g^− 1^ (Fig. 8a), which were lower than those of MB35 (212.23 F g^− 1^) and MB60 (289.14 F g^− 1^) when investigated at the mass loading of 1 mg cm^− 2^. After 5000 charge/discharge cycles, the specific capacitance of MB35 remained at 84.3% while that of MB60 remained at 65.4% with the Coulomb efficiency maintained at approximately 98% at 1 A g^− 1^ as shown in Fig. 8b. In addition, SEM images at different magnifications after 5,000 cycles of MB35 (**Fig. S6**) also showed that the surface structure of the electrode was still maintained stably, the conductive network and ion diffusion channel were still preserved. This demonstrated that the long-term cycling process did not cause serious morphological degradation, indicating sustainability in practical applications under high-load mass conditions. The electrochemical impedance spectra of MB35 and MB60 before and after 5,000 cycles at high load are shown in Fig. 8c. The results showed that MB60 exhibited a more obvious increase in R_ct_ from 5 to 27.55, while R_ct_ of MB35 increased from 4.19 to 8.25 after 5,000 cycles. In addition, UV–Vis analyses (Fig. 8d) showed that the MB concentration in the supercapacitor devices degraded significantly after 5000 cycles with degradation efficiencies reaching 99.76% for MB35 and 96.49% for MB60 (**Table S5**). Again, MB35 showed an optimal balance between electrochemical performance and cycling durability, thus being considered the most suitable configuration for the development of supercapacitor devices.

## Conclusion

Charcoal was synthesized via a simple ultrasonic treatment and employed as the active material for supercapacitor electrodes. To enhance the electrochemical performance of the charcoal-based device, methylene blue was introduced as a redox-active additive into an aqueous sodium chloride electrolyte. The incorporation of methylene blue, particularly at a concentration labeled MB35, significantly improved device performance by introducing additional pseudocapacitive charge storage through reversible redox reactions. This dual contribution, from electric double-layer capacitance and redox-mediated pseudocapacitance, led to a substantial enhancement in specific capacitance, energy density, and rate capability. The MB35-based symmetric supercapacitor demonstrated excellent cycling stability, retaining 91.3% of its initial specific capacitance after 2000 charge–discharge cycles at a specific current of 1.0 A g^–1^. It also achieved a high energy density of 15.34 Wh kg^–1^ at a power density of 350 W kg^–1^, approximately four times higher than the 3.70 Wh kg^–1^ delivered by the device without methylene blue under the same conditions. This work not only demonstrates a cost-effective and scalable approach to fabricating high-performance supercapacitors from biomass-derived carbon, but also highlights the multifunctional role of methylene blue. By enhancing electrochemical performance and simultaneously addressing environmental concerns related to dye pollution, this strategy offers a promising pathway toward sustainable, dual-purpose energy storage systems.

## Supplementary Information

Below is the link to the electronic supplementary material.

Supplementary Material 1

## Data Availability

Data sets generated during the current study are available from the corresponding author on reasonable request.
