# Supplementary material for: Methylene blue as a redox additive in electrolytes for advanced charcoal-based hybrid supercapacitors
Source: Sci Rep. 2025 Nov 6;15:38948. doi: 10.1038/s41598-025-22861-y (PMC12592482; doi:10.1038/s41598-025-22861-y)
Supplement: Supplementary file 1 — Supplementary Material 1 [file 41598_2025_22861_MOESM1_ESM.docx]

Methylene Blue as a Redox Additive in Electrolytes for Advanced Charcoal-Based Hybrid Supercapacitors

*Van Nhat Nguyen ^a^, An-Giang Nguyen ^a b^****,*** *Thi Viet Bac Phung ^a b^, Phi Long Nguyen ^a b^**

^a^ Center for Environmental Intelligence, VinUniversity, Hanoi 100000, Vietnam.

^b^ College of Engineering & Computer Science, VinUniversity, Hanoi 100000, Vietnam.

*Corresponding author: Phi Long Nguyen; E-mail: [long.np2@vinuni.edu.vn](mailto:long.np2@vinuni.edu.vn)

## **Table S1**.

| **Scan rate**  (mV s^–1^) | **Specific capacitance** (F g^–1^) | | | | |
| --- | --- | --- | --- | --- | --- |
|  | **MB0** | **MB5** | **MB10** | **MB35** | **MB60** |
| 5 | 83.62 | 161.98 | 157.34 | 274.57 | 348.15 |
| 10 | 77.55 | 133.64 | 129.56 | 217.66 | 255.80 |
| 20 | 71.40 | 112.37 | 110.87 | 167.84 | 185.89 |
| 40 | 63.41 | 93.32 | 93.86 | 127.08 | 133.92 |
| 60 | 56.87 | 80.13 | 83.71 | 105.28 | 109.81 |
| 80 | 51.36 | 69.30 | 74.25 | 89.99 | 95.30 |
| 100 | 46.45 | 60.55 | 66.67 | 75.51 | 83.50 |

## **Table S2**.

| **Specific current**  (A g^–1^) | **Specific capacitance** (F g^–1^) | | | | |
| --- | --- | --- | --- | --- | --- |
|  | **MB0** | **MB5** | **MB10** | **MB35** | **MB60** |
| 0.5 | 83.59 | 142.26 | 177.05 | 288.08 | 371.45 |
| 1 | 76.90 | 126.19 | 149.90 | 232.79 | 201.56 |
| 2 | 67.16 | 111.83 | 131.19 | 187.08 | 113.81 |
| 3 | 59.20 | 101.90 | 118.99 | 159.07 | 81.22 |
| 4 | 51.84 | 93.61 | 108.66 | 138.67 | 63.38 |
| 5 | 45.27 | 86.82 | 101.34 | 123.10 | 63.28 |
| 6 | 39.52 | 80.98 | 94.14 | 109.84 | 53.55 |
| 7 | 34.24 | 78.71 | 88.64 | 99.68 | 46.48 |
| 8 | 30.43 | 66.87 | 84.33 | 90.35 | 41.27 |
| 9 | 27.36 | 66.46 | 82.53 | 82.48 | 37.75 |
| 10 | 24.25 | 78.07 | 88.87 | 71.62 | 36.45 |

## **Table S3**.

| **Scan rate**  (mV s^–1^) | **Specific capacitance** (F g^–1^) | | | | |
| --- | --- | --- | --- | --- | --- |
|  | **MB0** | **MB5** | **MB10** | **MB35** | **MB60** |
| 5 | 110.48 | 123.95 | 134.92 | 292.74 | 317.27 |
| 10 | 94.51 | 99.16 | 116.00 | 298.93 | 272.37 |
| 20 | 72.16 | 82.08 | 99.02 | 227.40 | 227.77 |
| 40 | 50.31 | 66.81 | 81.53 | 174.47 | 186.56 |
| 60 | 39.66 | 57.78 | 71.11 | 147.30 | 162.68 |
| 80 | 33.43 | 51.05 | 63.81 | 129.14 | 146.50 |
| 100 | 29.17 | 45.92 | 58.28 | 115.32 | 134.93 |

## **Table S4**.

| **Specific current**  (A g^–1^) | **Specific capacitance** (F g^–1^) | | | | |
| --- | --- | --- | --- | --- | --- |
|  | **MB0** | **MB5** | **MB10** | **MB35** | **MB60** |
| 0.5 | 54.29 | 82.33 | 104.43 | 212.23 | 289.14 |
| 1 | 33.62 | 57.14 | 77.90 | 165.62 | 254.61 |
| 2 | 18.67 | 32.38 | 52.19 | 139.27 | 187.43 |
| 3 | 13.14 | 22.29 | 39.14 | 126.06 | 158.86 |
| 4 | 9.52 | 16.38 | 31.24 | 112.86 | 138.67 |
| 5 | 7.62 | 12.38 | 26.19 | 101.76 | 122.38 |
| 6 | 6.29 | 9.71 | 22.86 | 93.14 | 108.57 |
| 7 | 5.33 | 8.00 | 20.00 | 82.67 | 95.33 |
| 8 | 4.57 | 6.10 | 17.52 | 73.90 | 83.81 |
| 9 | 3.43 | 4.29 | 15.43 | 66.00 | 73.71 |
| 10 | 1.90 | 2.86 | 13.33 | 59.05 | 66.67 |

## **Table S5**.

| **Sample** | **The initial absorbance at 663 nm – A_0_** | **The absorbance at 663 nm after 5000 cycles - A_t_** | **Degradation efficiency** (%) |
| --- | --- | --- | --- |
| **MB35** | 1.55933 | 0.00367 | 99.76 |
| **MB60** | 2.151 | 0.07533 | 96.49 |

## **Table S6**.

| **Sample** | **Initial pH** | **pH After 5000 cycles** |
| --- | --- | --- |
| **MB0** | 6.48 |  |
| **MB5** | 5.92 |  |
| **MB10** | 5.81 |  |
| **MB35** | 5.38 | 7.19 |
| **MB60** | 4.69 | 7.08 |


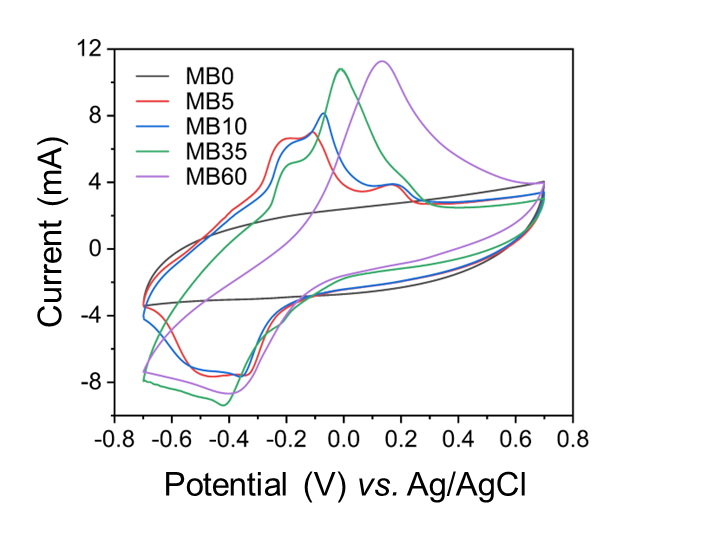


# **Fig. S1**. Comparison of CV curves of all samples at a scan rate of 100 mV s^–1^ in the three-electrode system.


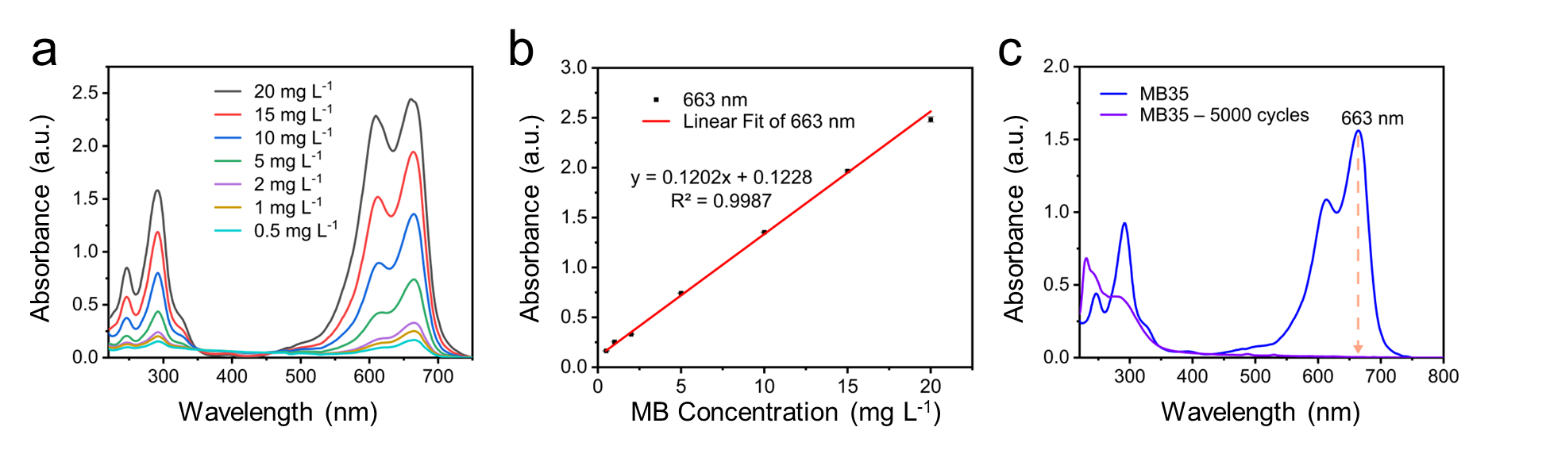


# **Fig. S2**. (a) UV-Vis spectrum; (b) Calibration curve of MB solution between 0.5 and 20 mg L^-1^.


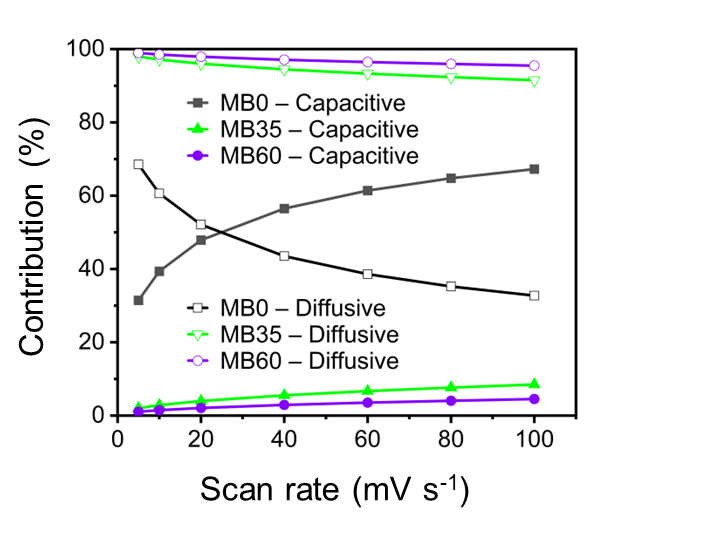


# **Fig. S3.** Capacitive and diffusive contributions to overall charge storage of MB0, MB35 and MB60 at various scan rates.


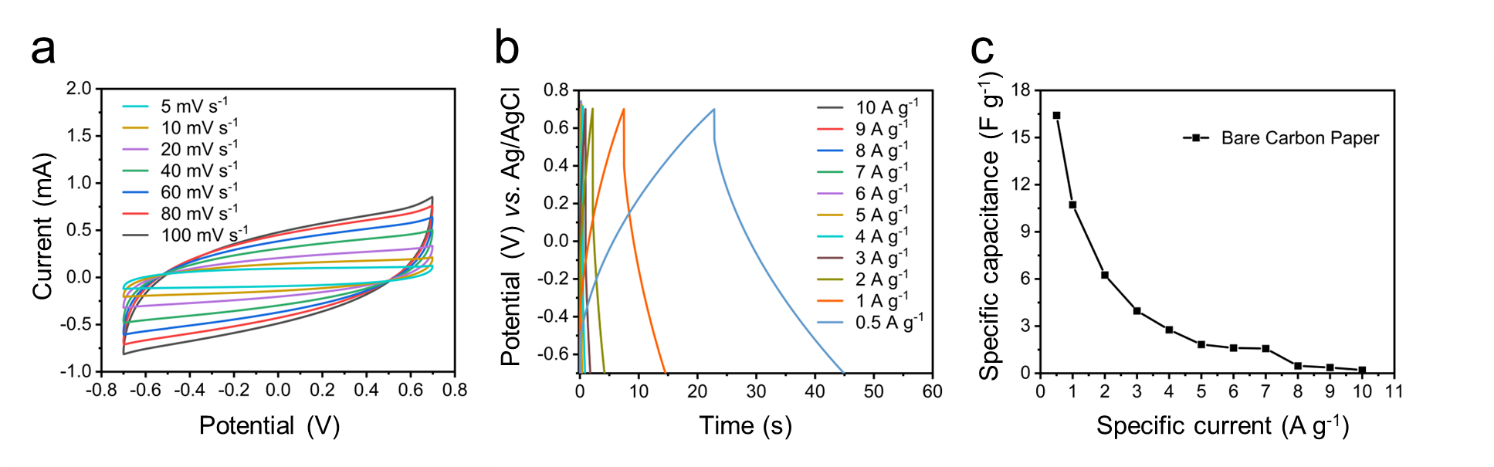


# **Fig. S4.** (a) CV curves; (b) Galvanostatic charge–discharge curves, and (c) Specific capacitances at various specific current of the carbon paper electrode in the three-electrode system.


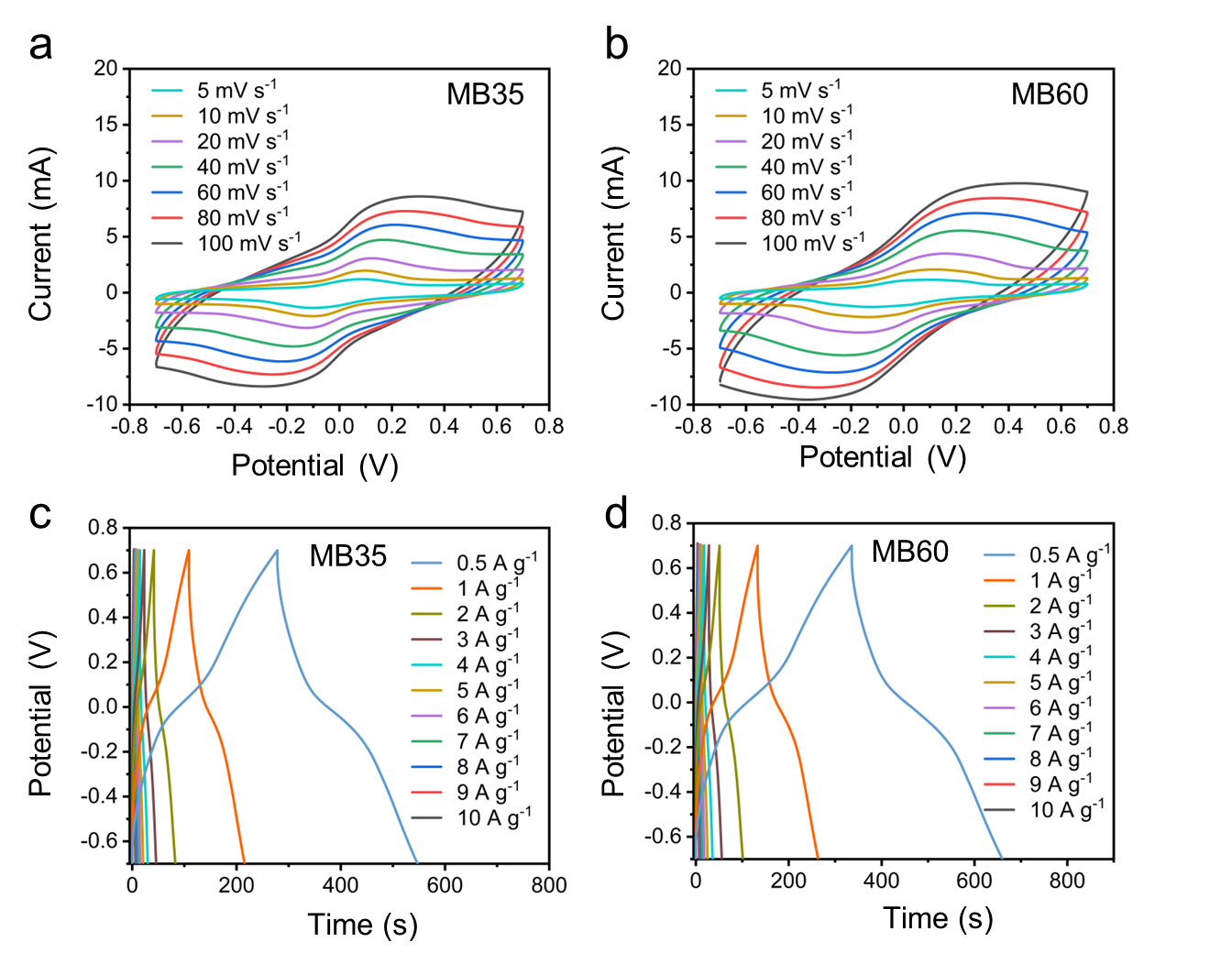


# **Fig. S5.** CV curves at various scan rates and Galvanostatic charge–discharge curves at various specific currents for the symmetrical supercapacitor device at the high loading of 2 mg cm^-2^: (a, c) MB35, (b, d) MB60.


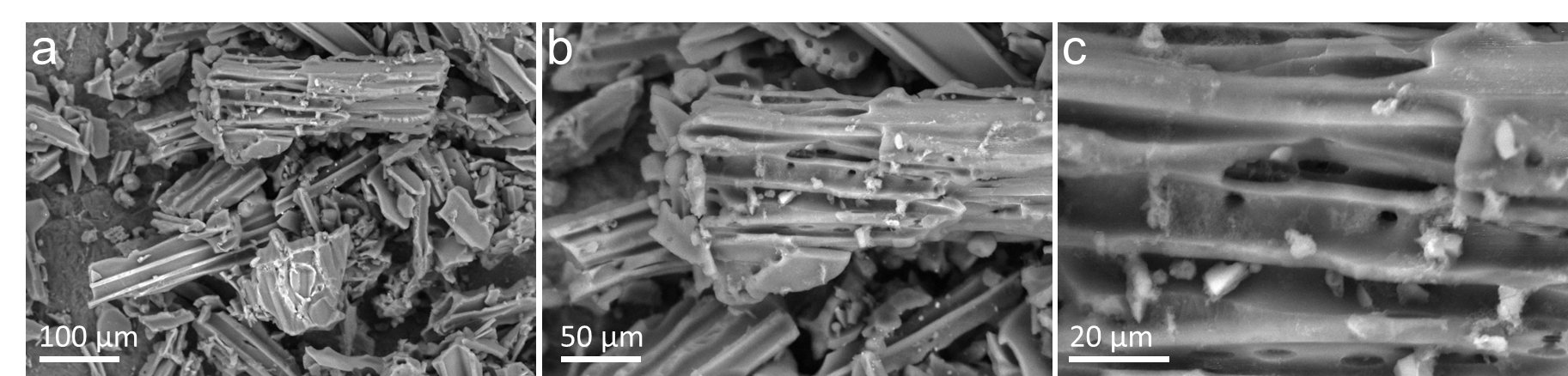


# **Fig. S6.** SEM images of the electrode material recorded under various magnifications after 5,000 cycles.


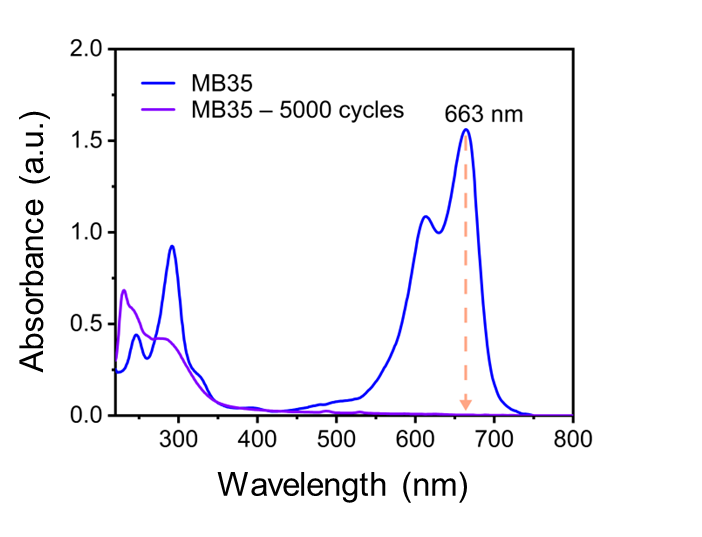


# **Fig. S7.** Evolution of the UV–Vis absorption spectra of the electrolyte solution of MB35 after 5000 cycles.
